# Testing the Island Effect in a Highly Mobile Pollinator: Wing Morphological Divergence in *Euglossa mixta* from Continental and Insular Panama

**DOI:** 10.3390/ani16020227

**Published:** 2026-01-12

**Authors:** Yostin Añino, Jordan Hernández-Martelo, Fernando Moya, Alejandro Piñeiro-González, Laura M. Pérez, Dumas Gálvez, Yosiat Vega-Rovira, Julio Trujillo, Anette Garrido, Danilo Arrocha, Franco Cruz-Jofré, Hugo A. Benítez

**Affiliations:** 1 Museo de Invertebrados G.B. Fairchild, Universidad de Panamá, Ciudad de Panamá 0824-0021, Panama; yostin0660@gmail.com (Y.A.); anecgarrido@gmail.com (A.G.); danilo.arrocha1881@gmail.com (D.A.); 2 Coiba Scientific Station, City of Knowledge, Clayton, Panamá City 0843-01853, Panama; dgalvez@coiba.org.pa; 3 Laboratorio de Ecología y Morfometría Evolutiva, Instituto One Health, Facultad de Ciencias de la Vida, Universidad Andrés Bello, República 440, Santiago 8370134, Chile; 4 Programa de Doctorado en Salud Ecosistémica, Centro de Investigación de Estudios Avanzados del Maule, Universidad Católica del Maule, Talca 3466706, Chile; 5 Millennium Institute Biodiversity of Antarctic and Subantarctic Ecosystems (BASE), Santiago 7800003, Chile; fernando.moya@ug.uchile.cl (F.M.); francocruzjo@santotomas.cl (F.C.-J.); 6 Cape Horn International Center (CHIC), Centro Universitario Cabo de Hornos, Puerto Williams 6350000, Chile; 7 Laboratorio de Ecología Molecular, Departamento de Ciencias Ecológicas, Facultad de Ciencias, Universidad de Chile, Santiago 7800003, Chile; 8 Vicerrectoría de Investigación y Postgrado, Universidad de La Serena, La Serena 1700000, Chile; andresale96@gmail.com; 9 Departamento de Ingeniería Industrial y de Sistemas, Universidad de Tarapacá, Arica 1000000, Chile; lperez@uta.cl; 10 Programa Centroamericano de Maestría en Entomología, Universidad de Panamá, Ciudad de Panamá 0824-0021, Panama; yosiat13@gmail.com; 11 Sistema Nacional de Investigación (SNI), Panamá City 0816-02852, Panama; 12 Departamento de Matemática, Facultad de Ciencias Naturales, Exactas y Tecnología, Universidad de Panamá, Ciudad de Panamá 0824-0021, Panama; julio.trujillo@up.ac.pa; 13 Programa de Doctorado en Matemática Aplicada, Facultad Regional Multidisciplinaria de Chontales, Universidad Nacional Autónoma de Nicaragua, Managua 14172, Nicaragua; 14 Centro de Investigación de Operaciones para el Desarrollo, la Ciencia y la Tecnología, Universidad de Panamá, Ciudad de Panamá 0824-0021, Panama; 15 Facultad de Recursos Naturales y Medicina Veterinaria, Universidad Santo Tomás, Viña del Mar 2520000, Chile; 16 Centro de Investigación de Estudios Avanzados del Maule, Universidad Católica del Maule, Talca 3530000, Chile

**Keywords:** wing shape, pollinators, geometric morphometrics, Mahalanobis distances, Island effect

## Abstract

Islands provide valuable opportunities to study how isolation affects phenotypic variation. Even though orchid bees are highly mobile, their movement can still be restricted by marine barriers. In this study, we assessed whether insular isolation impacts wing shape in the orchid bee *Euglossa mixta* across the Coiba archipelago and a nearby mainland site in Western Panama. Our study analyzed 271 individuals using geometric morphometrics, focusing on forewing venation landmarks, and evaluated the variation using multivariate analyses of shape variation and quantifying the shape of Mahalanobis distances. Additionally, we conducted a Mantel test to explore the relationship between geographic distance and morphological divergence. Our findings reveal that wing shape variation in *E. mixta* is largely conserved but shows fine-scale structuring consistent with spatial patterns expected in insular systems. These results suggest that even highly mobile pollinators may experience enough isolation for subtle phenotypic shifts to occur, highlighting the sensitivity of geometric morphometrics for detecting early stages of morphological differentiation.

## Simple Summary

Islands provide unique opportunities to explore how isolation affects the lives of organisms. Although orchid bees are strong fliers, which leads to the assumption that their mobility prevents significant population differences, our study examined the wing shape of the orchid bee *Euglossa mixta* from the Coiba archipelago islands and a nearby mainland site in Panama. By using geometric morphometrics, we identified subtle yet consistent differences in wing shape across different sites. These variations were more pronounced between the distant islands and the mainland, indicating that even highly mobile pollinators can develop fine-scale morphological differences in insular environments.

## 1. Introduction

The morphological variation in organisms is influenced by both genetic factors and the environmental and ecological conditions under which their development occurs [1,2]. In particular, insular populations provide an ideal framework for studying these processes, as they represent a major source of evidence for the occurrence of rapid adaptive changes in morphological traits [3,4]. The inherent properties of islands, such as geographic isolation, small population sizes, and limited resource availability, among others, contribute to the isolation of evolutionary processes that promote local adaptation and, over time, speciation, a phenomenon commonly referred to as the island effect [5,6,7,8,9]. Consequently, populations inhabiting islands often exhibit phenotypic modifications compared to their continental counterpart, as a result of specific selective pressures associated with resource availability, intraspecific competition, dispersal constraints, or the absence of predators [10,11]. These changes may be expressed through variation in body size, wing proportions, locomotor structures, or feeding-related traits, reflecting the plasticity and adaptive response of species to insular environmental conditions [12,13].

Beyond their biogeographic isolation, islands function as natural laboratories for evolutionary experimentation, where selective pressures operate with greater intensity or independence than in continental systems [8,14]. As a result, insular environments provide valuable opportunities to observe the tempo and mode of evolutionary change, revealing how ecological constraints, resource limitation, and spatial isolation interact to shape phenotypic diversity [3,15]. In this sense, morphological variation serves as a key proxy for understanding the adaptive trajectories and diversification processes that characterize island biotas.

Although the particular conditions of islands may promote phenotypic divergence and the emergence of local adaptations, certain evolutionary processes can, in some cases, lead to the opposite effect by reducing genetic variability and, consequently, morphological variation [16,17]. Factors such as reduced gene flow, recent colonization events, founder effects, and genetic drift tend to limit diversity within insular populations [18,19]. Moreover, when islands are located close to one another or share similar environmental conditions, a certain degree of morphological homogenization may be maintained as a result of the occasional exchange of individuals or partial panmixia [20,21]. Taken together, these processes indicate that insularity does not always promote morphological differentiation; rather, its effect depends on the interaction between the evolutionary history of populations, the degree of connectivity among islands, and local selective pressures.

In this context, several studies have documented patterns of morphological variation across different taxa [22,23,24,25]; however, such processes have been scarcely explored in insular insects [7]. Insects represent a particularly suitable model for studying the effects of insularity on shape, given their wide distribution, high dispersal capacity, and sensitivity to local environmental conditions [7,26,27]. Among these, *Euglossa mixta* Friese, 1899 stands out as a valuable model species. This bee belongs to the tribe Euglossini, which includes approximately 200–250 species commonly known as orchid bees [28,29]. These bees are native to the Neotropics and are distinguished by their bright metallic coloration, their importance as bioindicators, and the behavior of males, which collect volatile chemical compounds from a wide range of sources, including orchids, but not limited to floral ones [9,30]. Their importance in pollination and their role as bioindicators of ecosystem health, as reflected by changes in composition and abundance, combined with the presence of populations both on the mainland and across oceanic island complexes, make *E. mixta* an ideal model for exploring how geographic isolation and environmental conditions shape morphology [31,32,33]. This species, therefore, provides an opportunity to test whether classical insular patterns, such as reduced variability or trait shifts associated with isolation, are also expressed in highly mobile pollinators. Because our goal is to test for subtle morphological responses to insularity, an analytical framework that captures fine-scale shape differences is required. In this context, geometric morphometrics provides an ideal analytical framework for assessing subtle morphological variation in *E. mixta*, as it allows the precise quantification and comparison of biological shapes. By capturing the geometry of anatomical structures through the placement of homologous landmarks, this approach enables the detection of fine-scale shape differences [34,35]. Wing venation variability is not uniform across Apoidea, with some tribes, such as Eucerini, exhibiting markedly higher morphological plasticity than Euglossini. In contrast, the relatively conserved wing architecture of orchid bees makes the detection of population-level shape differences particularly informative, as it reduces the likelihood that observed variation reflects taxon-specific instability rather than spatial structuring.

This methodology enables the capture of the complete geometry of structures, providing a detailed and statistically rigorous description of morphological variation patterns [36,37,38,39].

In insects, wing structures have been widely used in studies of population differentiation and adaptation, as they reflect both genetic components and phenotypic responses to environmental conditions [40]. For example, Ostwald et al. [7] evaluated variation in the wing venation patterns of the social bee *Halictus tripartitus* Cockerell, 1895, comparing an isolated population from Santa Cruz Island (California) with mainland populations, and found significant morphological differentiation between the two groups. Similarly, Laojun et al. [6] analyzed wing size and shape in the mosquito vectors *Aedes albopictus* (Skuse, 1894) and *Armigeres subalbatus* (Coquillett, 1898) across the Ranong and Trat archipelagos (Thailand), reporting significant differences in centroid size, used as a proxy for body size, and slight variations in wing shape. These studies highlight that wing morphology, even in closely related or geographically proximate populations, can reveal evolutionary responses to isolation and local adaptation.

Western Panama constitutes an exceptional natural laboratory for investigating the effects of insularity on morphological variation. This region is characterized by its high biological diversity, elevated endemism, and complex geographical configuration [41,42]. In particular, the Coiba Island National Park System, composed of Coiba Island and 38 smaller islands (including Coibita, Jicarón, and Canales) located in the Gulf of Chiriquí, provides a unique opportunity to evaluate processes of rapid morphological adaptation driven by gradients of isolation and environmental conditions that contrast with those of the mainland [9,43]. The archipelago presents gradients of humidity, temperature, and vegetation cover that may influence flight performance and resource availability, providing an environmental context in which subtle variation in wing shape could arise. Although the bee fauna of the Coiba archipelago has been increasingly documented in recent years, information on population-level structuring and morphological variation across islands remains limited, particularly for highly mobile pollinators. This context is especially relevant for species with high dispersal capacity, such as *E. mixta*, whose insular and continental populations allow for the exploration of the relationship between geographic isolation and phenotypic differentiation.

*Euglossa mixta* is a relatively large-bodied orchid bee with strong flight capacity and broad foraging ranges, traits that make it a conservative and biologically informative model for testing insular effects. If morphological differentiation is detectable in a species with high dispersal ability and constrained wing venation, such patterns are unlikely to reflect trivial isolation and instead point to subtle but consistent spatial structuring. Moreover, *E. mixta* is one of the few orchid bee species consistently recorded across both the mainland and multiple islands of the Coiba archipelago, allowing comparisons across a well-defined gradient of geographic isolation.

Within this framework, this study aims to quantify variation in the wing morphology of *E. mixta* between insular and continental populations to assess whether geographic isolation influences phenotypic differentiation. Using a geometric morphometric approach, it seeks to determine whether distance and insular isolation are reflected in detectable changes in wing shape, providing evidence for the role of insularity in shaping the morphological structure of this Neotropical species. Therefore, this study hypothesizes that greater geographic isolation is associated with detectable divergence in wing shape, reflecting the influence of insular conditions on morphological differentiation.

## 2. Materials and Methods

**Figure 1:**
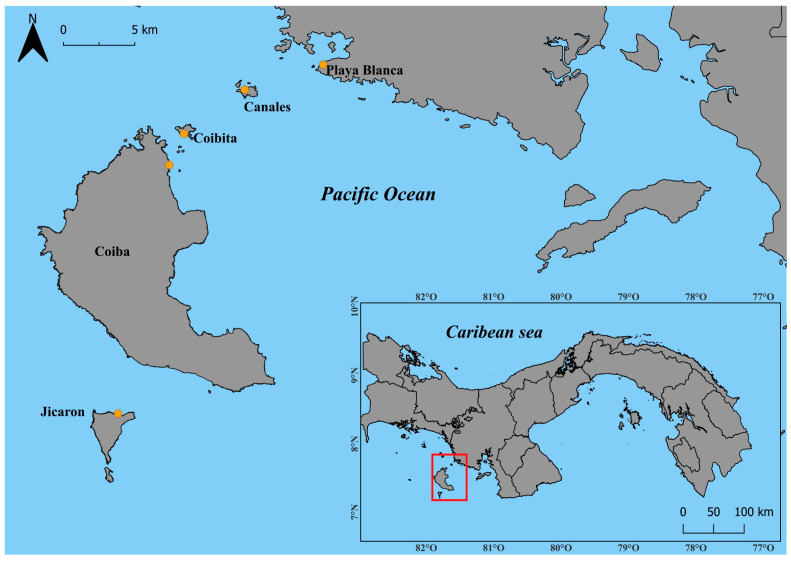
Geographic location of the study area in Western Panama, showing the Coiba archipelago and mainland sampling sites. The inset map highlights the position of the archipelago within the country.

To quantify the morphological variation through shape analysis in *E. mixta* across the mainland and different islands, 271 male individuals of *E. mixta* were collected during May and June 2023 using modified traps containing a chemical attractant compound Rigar^®^ Balsam (Laboratorio Rigar S.A., Panamá, Panama). All specimens were described in detail in Vega-Rovira. et al. [9]. Specimens were identified using the taxonomic keys of Roubik and Hanson (2004) [44] and were preserved in 96% ethanol for subsequent processing. Five sites were sampled (Figure 1), Playa Banca (PYB) in the mainland (7°43′33.47″ N, 81°31′45.758″ W) (n = 12), and four islands that varied in area and distance from the mainland: Canales de Afuera (CAN) (7°41′40.995″ N, 81°37′44.651″ W) (n = 26), Coibita (CBI) (7°38′22.062″ N, 81°42′22.057″ W) (*n* = 76), Coiba (CBA) (7°36′1.89″ N, 81°43′28.76″ W) (n = 78) and Jicarón (JIC) (7°17′22.7040″ N, 081°47′22.4520″ W) (n = 79).

**Figure 2:**
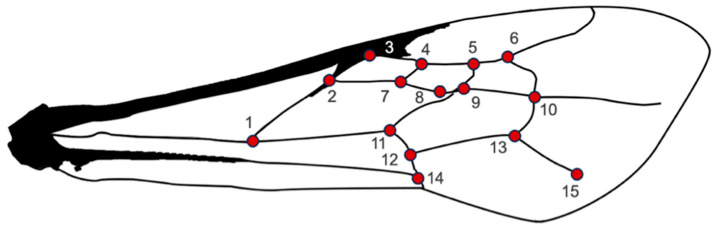
Forewing configuration of *Euglossa mixta*, showing the 15 landmarks used to characterize wing shape.

The right forewings were carefully detached, cleaned of excess tissue, air-dried, and mounted flat on microscope slides prior to imaging. The wings preserved on microscope slides are deposited in the G. B. Fairchild Invertebrate Museum, following an alphanumeric registration code that includes locality–individual–wing. Wings were photographed using a stereomicroscope with a calibrated scale. Images were organized into a .tps file using tpsUtil v1.81 [45]. For each wing, 15 landmarks were digitized using the software tpsDig2 v2.31 [45]. The landmarks were placed at the intersections of the wing venation (Figure 2), and their information was extracted by a Procrustes fit, which standardizes the samples, eliminating the effect of size, position and rotation (Rohlf and Slice, 1990) [37].

In order to visualize the wing shape variation, a Principal Component Analysis (PCA) was performed using the Covariance Matrix of Procrustes-aligned coordinates [46]. On the other hand, to reduce the number of shape dimensions and maximize the differentiation between groups (sites), new shape axes were created performing a Canonical Variate Analysis (CVA) [47]. CVA was used as an exploratory tool to maximize between-group shape differences and visualize patterns of population-level structuring, rather than as a predictive classification approach. To statistically evaluate the differences in shape between sites, a permutation test of 10,000 rounds was conducted using the Mahalanobis distances (distances or magnitude of difference between average shapes) extracted from the CVA. Geometric morphometrics analyses were conducted through the software MorphoJ v1.07 [48] and the package “geomorph V. 4.0.10” in R software v 4.04 [49]. 

Finally, to evaluate whether differences in the shape of the forewings of *E. mixta* are associated with geographic distances between islands and the mainland, a Mantel test [50] was conducted among sampling points (sites), comparing a matrix of geographic distances with a matrix of Mahalanobis shape distances. The shape distances were extracted from CVA, and the geographical distances between sites were obtained from QGIS software v3.20.2.

## 3. Results

**Figure 3:**
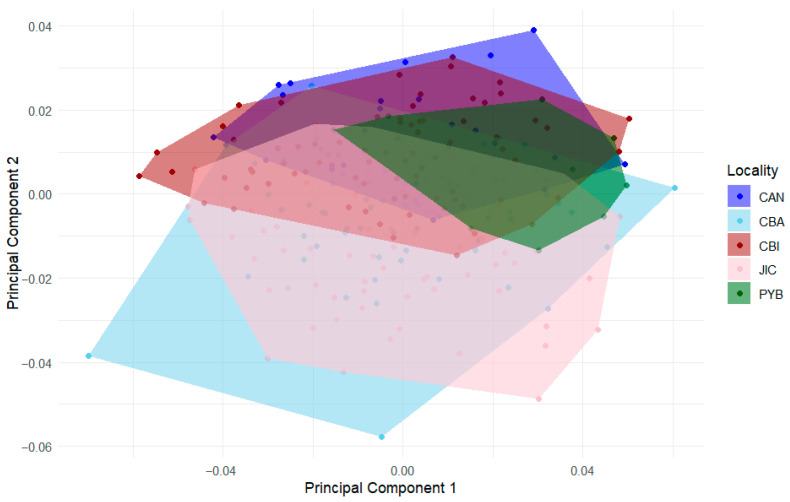
Principal Component Analysis (PCA) of the right forewing of *Euglossa mixta*. Points represent individual specimens, and polygons indicate the convex hulls for each locality. Both points and convex hulls are colored according to locality: Canales (CAN), Coiba (CBA), Coibita (CBI), Playa Blanca (PYB), and Jicarón (JIC).

The Principal Component Analysis (PCA) of the wing shape shows that 33.1% of the variance is explained by the first two components (PC1 = 22.6%; PC2 = 10.5%). The morphospace of these two principal components shows an overlap in the shape of all sites, with a higher variation in the shape along principal component two (Figure 3).

**Figure 4:**
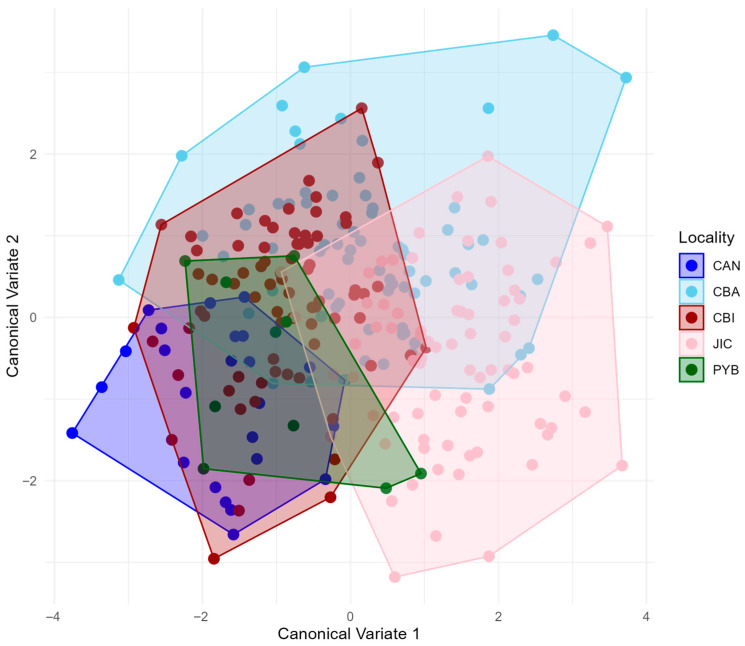
Canonical Variate Analysis of the five *E. mixta* sampling sites. Points and convex hulls are colored according to the locality: Canales (CAN), Coiba (CBA), Coibita (CBI), Playa Blanca (PYB), and Jicarón (JIC).

In contrast, the Canonical Variate Analysis shows a subtle differentiation between sites, varying mainly throughout the canonical variate one, and separating the samples into groups composed of Canales, Coibita and Playa Blanca. The two remaining sites (Jicaron and Coiba) individually share a part of their morphospace between them and with the main group (Figure 4).

**Figure 5:**
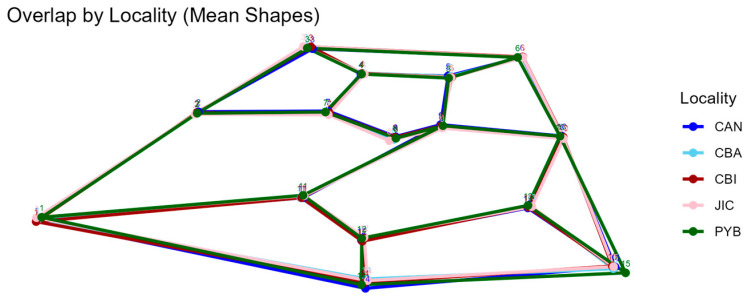
Graphical representation of the superposition of the average forewing shape of the five sampled sites used in this study: Playa Blanca (PYB) (green), Canales de Afuera (CAN) (blue), Coiba (CBA) (light blue), Coibita (CBI) (red) and Jicaron (JIC) (pink).

The superposition of mean wing shapes (Figure 5) reveals subtle but consistent spatial shifts in landmark positions among sites. The most noticeable differences involve the mainland population (Playa Blanca), which shows slight positional displacements relative to insular populations at landmarks associated with vein intersections and inflection points, such as landmark 15. Similarly, landmarks 6 and 1 exhibit small but coherent shifts in position in the Playa Blanca samples compared to the islands. These differences reflect localized changes in wing vein geometry and curvature rather than direct variation in vein length, as geometric morphometric analyses capture relative landmark displacement within the overall wing configuration.

**Figure 6:**
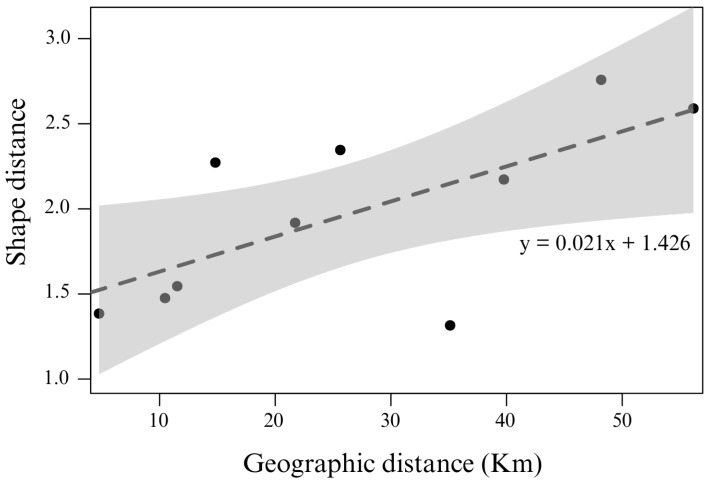
Scatter plot of the Mantel test between the shape distances matrix (as Mahalanobis shape distance) and the geographical distances matrix. The trend line is fitted to a linear model. The gray band shows the 95% confidence interval.

Regarding the shape differences, the permutation test was conducted between the Mahalanobis shape distances, which showed a significant difference in the forewing shape between all sites, except for the wing between individuals of *E. mixta* from Playa Blanca and Canales de Afuera (Table 1). The highest shape difference was between Jicaron and Canales de Afuera, while the least difference was between Coiba and Jicaron. Finally, the Mantel test shows a positive and significant correlation between the geographical and the Mahalanobis shape distances (r = 0.64; *p* < 0.05), suggesting that individuals from the most distant sites exhibit higher differentiation in wing shape, while sites closer together have individuals with more similar wing shapes (Figure 6). One pairwise comparison deviates from the overall trend, which is expected given the limited number of sampling sites, and does not affect the significance of the Mantel correlation.

## 4. Discussion

Beyond their geographic boundaries, islands represent powerful natural laboratories that amplify the effects of spatial structure on evolutionary processes. Islands provide particularly valuable settings for examining evolutionary change because their isolation restricts gene flow among populations and creates opportunities for divergence to accumulate over time [14]. Under such conditions, genetic drift can exert a stronger influence on phenotypic patterns, especially in small or partially isolated populations [51]. These stochastic forces often interact with local ecological pressures, making archipelagos effective arenas for understanding how morphology evolves under limited gene flow and heterogeneous environmental contexts. Geometric morphometrics has proven an effective tool for addressing our central question: whether insular isolation influences wing shape in *E mixta*. This approach captures small but biologically meaningful variations in shape that traditional linear measurements are less likely to detect [36]. In our dataset, the ability of geometric morphometrics to retain spatial relationships among wing-vein landmarks was essential for uncovering subtle phenotypic differences among populations.

Although the differences observed were subtle, the resulting patterns provide valuable insights into the natural history of *E. mixta*. In bees, wing venation is a highly conserved trait shaped by aerodynamic demands, developmental constraints, and phylogenetic history, providing mechanical support for flight while allowing limited but informative variation among populations and species (e.g., Cameron [28], Perrard et al. [52]). In Euglossini, despite their strong flight capacity, subtle variation in wing shape and venation has been shown to reflect population-level structuring rather than major functional shifts.

Orchid bees are recognized for their strong flying abilities, wide foraging ranges, and capacity to navigate varied landscapes [28]. These traits often minimize the likelihood of population differentiation in fragmented or isolated systems. Previous studies have shown that high mobility can buffer the effects of isolation by maintaining gene flow across spatially structured environments [19,20]. Under these conditions, morphological divergence among islands would not be expected. Therefore, the consistent shape differences observed here indicate that insular conditions impose ecological or spatial constraints strong enough to generate localized phenotypic changes. This pattern suggests that morphological variation in *E. mixta* results from an interaction between the regulating effects of dispersal and the diversifying pressures associated with geographic isolation, resource structure, and the environmental characteristics unique to each island.

Patterns of subtle yet statistically robust morphological divergence, such as those observed in *E. mixta*, have also been reported in other insular insect systems examined using geometric morphometrics. For example, Laojun et al. [6] found that populations of *Aedes albopictus* and *Armigeres subalbatus* in the Thai archipelagos showed only partial separation in canonical space, while exhibiting significant Mahalanobis distances between the islands. These findings align with ours, showing that morphological divergence in island insects often results in subtle rather than substantial changes. Similar patterns have been observed in *Praocis spinolai* (Solier, 1841) from coastal Chile, for which Benítez et al. [27] demonstrated that island populations exhibit subtle but consistent differences in traits related to locomotion and body robustness compared to their mainland counterparts. In the island–mainland comparison of the halictid bee *Halictus tripartitus*, Ostwald et al. [7] found consistent differentiation in wing venation, despite significant overlap in morphospace. This indicates that population-specific characteristics can emerge even in species with high dispersal potential. Together, these studies support the idea that islands often encourage fine-scale morphological changes in insects. Additionally, geometric morphometrics proves particularly effective for identifying these patterns, even in species with relatively high mobility. There is a strong positive correlation between geographic distance and differences in wing shape, further supporting the idea that spatial isolation influences the phenotypic variation in *Euglossa mixta.* This pattern aligns with the isolation-by-distance dynamics often observed in insular species, in which even slight reductions in connectivity can lead to noticeable divergence in morphological traits [5].

The differences observed between the most remote island, Jicarón, and the other locations indicate that dispersal between distant islands is either rare or inadequate to fully equalize wing shape. Similar distance-related patterns have been noted in other island-dwelling insects. This pattern aligns with the dynamics of isolation-by-distance that are often observed, as reported by Laojun et al. [6]. They found that the largest Mahalanobis distances, when comparing insular Hymenoptera with mainland populations, occurred between populations separated by wider marine barriers. Similar patterns have also been observed in studies of non-native species, where gradual divergence can occur even in the presence of ongoing gene flow among localities. For example, spatial differentiation has been detected in *Trichocera maculipennis* Meigen, 1830 across Antarctic stations [23], with greater wing shape divergence observed at the geographically most isolated locality, separated by Fildes Bay and the Collins Glacier.

Although geographic isolation is a primary factor in shaping wing shape in *E. mixta*, environmental variation between the islands could also contribute to the observed phenotypic patterns. Differences in vegetation structure, availability of floral resources, microclimatic conditions, and wind exposure can all influence flight behavior and aerodynamic demands, which could generate specific functional demands at the island level on wing morphology [53,54]. Similar environmentally driven variations have been documented in other insects, where local habitat conditions affect wing venation and flight dynamics [55,56]. In this context, the subtle but consistent differences observed among islands may indicate an interaction between spatial isolation and the performance demands imposed by the environment. Future studies that incorporate habitat descriptors or directly measure flight behavior would help clarify the extent to which local ecological conditions contribute to the morphological differentiation seen in *E. mixta*. Finally, our results show that *E. mixta* exhibits subtle but consistent morphological structuring across the Coiba archipelago, revealing how insular environments can shape phenotypic variation even in species with high dispersal capacity. Although the detected differences in wing shape are smaller, the agreement across multivariate analyses, the significant Mahalanobis distances, and the positive association between geographic distance and shape variation indicate that these patterns reflect more than sampling noise. Instead, they point to a landscape of partial connectivity in which both spatial isolation and local environmental conditions contribute to fine-scale morphological differentiation. While our data do not allow us to determine whether these shifts are genetically based or environmentally induced, the patterns are consistent with early-stage divergence observed in other island insects. In Hymenoptera, males are haploid, which may allow genetic differences among populations to be more directly expressed at the phenotypic level [57,58]. However, because our analyses rely exclusively on wing shape data, we cannot disentangle the relative contributions of genetic differentiation and phenotypic plasticity. Future work integrating genomic data, environmental variables and functional measures of flight performance will be essential for assessing the relative contributions of drift, selection and plasticity. Nonetheless, this study demonstrates that geometric morphometrics is a sensitive and informative tool for detecting subtle phenotypic divergence in *E. mixta*, providing a foundation for understanding how insular systems influence the evolutionary trajectories of highly mobile pollinators.

## 5. Conclusions

This study shows that the wing shape of *Euglossa mixta* remains consistent mainly across the Coiba archipelago and the mainland. However, subtle morphological variations have emerged due to insular isolation. These patterns suggest that even highly mobile pollinators can experience spatial constraints that allow for fine-scale differences in their traits. Instead of showing either substantial divergence or complete uniformity, the morphology of *E. mixta* represents a balance between its ability to disperse and the geographic layout of the island systems. This underscores how insular environments can influence pollinators’ functional traits, highlighting the interplay between mobility, environmental conditions, and spatial structures in shaping phenotypic variation. In the future, integrating genomic, ecological, and functional data will be critical for uncovering the mechanisms behind these patterns and evaluating their significance in the context of pollinator ecology and island biogeography.

## Figures and Tables

**Table 1:** Matrix of Mahalanobis shape distances (lower diagonal) and geographical distances (upper diagonal) between sampling sites for *E. mixta*. The shape distances are presented in Mahalanobis shape units (dimensionless), while geographical distances are presented in kilometers.

Locality	Canales	Coiba	Coibita	Jicaron	Playa Blanca
Canales		14.82	10.47	48.17	11.52
Coiba	2.2723		4.76	35.12	25.65
Coibita	1.4758	1.3848		39.76	21.72
Jicaron	2.7581	1.3153	2.1725		56.15
Playa Blanca	1.5458	2.3459	1.9182	2.5898	

## Data Availability

Data is available in the Supplementary Material file and further inquiries can be directed to the authors.

## Supplementary Materials

The following supporting information can be downloaded at: https://www.mdpi.com/article/10.3390/ani16020227/s1, The raw data is available as a supplementary data.

## References

[1] Kingsolver J.G., Huey R.B. (2003). Introduction: The evolution of morphology, performance, and fitness. Integr. Comp. Biol..

[2] Miner B.G., Sultan S.E., Morgan S.G., Padilla D.K., Relyea R.A. (2005). Ecological consequences of phenotypic plasticity. Trends Ecol. Evol..

[3] Losos J.B., Ricklefs R.E. (2009). Adaptation and diversification on islands. Nature.

[4] Thompson J.N. (1998). Rapid evolution as an ecological process. Trends Ecol. Evol..

[5] Emerson B.C. (2002). Evolution on oceanic islands: Molecular phylogenetic approaches to understanding pattern and process. Mol. Ecol..

[6] Laojun S., Sontigun N., Chaiphongpachara T. (2024). Influence of insular conditions on wing phenotypic variation in two dominant mosquito vectors, *Aedes albopictus* and *Armigeres subalbatus* (Diptera: Culicidae), in the border archipelagos of Thailand. Med. Vet. Entomol..

[7] Ostwald M.M., Thrift C.N., Seltmann K.C. (2023). Phenotypic divergence in an island bee population: Applying geometric morphometrics to discriminate population-level variation in wing venation. Ecol. Evol..

[8] Whittaker R.J., Fernández-Palacios J.M., Matthews T.J., Borregaard M.K., Triantis K.A. (2017). Island biogeography: Taking the long view of nature’s laboratories. Science.

[9] Vega-Rovira Y.A., Bennett-Vaz R., Araúz G., Añino Y., Gálvez D. (2025). Island Biogeography Theory and the Island Rule in Orchid Bees in Panama. J. Biogeogr..

[10] Jordan M.A., Snell H.L. (2008). Historical fragmentation of islands and genetic drift in populations of Galápagos lava lizards (*Microlophus albemarlensis* complex). Mol. Ecol..

[11] Souza F.S.d., Nunes L.A., Oliveira E.J.F.d., Costa M.A.P.d.C., Carvalho C.A.L.d. (2019). Population variation and island effect in *Melipona subnitida* (Hymenoptera: Apidae). J. Apic. Res..

[12] Kraemer A.C., Philip C.W., Rankin A.M., Parent C.E. (2019). Trade-offs direct the evolution of coloration in Galápagos land snails. Proc. Biol. Sci..

[13] Palkovacs E.P. (2003). Explaining adaptive shifts in body size on islands: A life history approach. Oikos.

[14] Warren B.H., Simberloff D., Ricklefs R.E., Aguilée R., Condamine F.L., Gravel D., Morlon H., Mouquet N., Rosindell J., Casquet J. (2015). Islands as model systems in ecology and evolution: Prospects fifty years after MacArthur-Wilson. Ecol. Lett..

[15] Losos J.B., Glor R.E., Kolbe J.J., Nicholson K. (2006). Adaptation, speciation, and convergence: A hierarchical analysis of adaptive radiation in Caribbean Anolis lizards1. Ann. Mo. Bot. Gard..

[16] Funk W.C., Lovich R.E., Hohenlohe P.A., Hofman C.A., Morrison S.A., Sillett T.S., Ghalambor C.K., Maldonado J.E., Rick T.C., Day M.D. (2016). Adaptive divergence despite strong genetic drift: Genomic analysis of the evolutionary mechanisms causing genetic differentiation in the island fox (*Urocyon littoralis*). Mol. Ecol..

[17] Recuerda M., Montoya J.C.H., Blanco G., Milá B. (2024). Repeated evolution on oceanic islands: Comparative genomics reveals species-specific processes in birds. BMC Ecol. Evol..

[18] Estoup A., Ravigné V., Hufbauer R., Vitalis R., Gautier M., Facon B. (2016). Is there a genetic paradox of biological invasion?. Annu. Rev. Ecol. Evol. Syst..

[19] Nicholson D.J., Knell R.J., Folfas E., Neel L.K., Degon Z., DuBois M., Ortiz-Ross X., Chung A.K., Curlis J.D., Thurman T.J. (2023). Island colonisation leads to rapid behavioural and morphological divergence in Anolis lizards. Evol. Ecol..

[20] Logan M.L., Duryea M.C., Molnar O.R., Kessler B.J., Calsbeek R. (2016). Spatial variation in climate mediates gene flow across an island archipelago. Evol. Int. J. Org. Evol..

[21] Yi C., Zheng C., Zeng L., Xu Y. (2016). High genetic diversity in the offshore island populations of the tephritid fruit fly Bactrocera dorsalis. BMC Ecol..

[22] Arriagada J.I., Benítez H.A., Toro F., Suazo M.J., Abarca P., Canto J., Vilina Y.A., Cruz-Jofré F. (2022). Insularity and Aridity as Drivers of Mandibular Disparity in *Thylamys elegans* (Waterhouse, 1839) from Populations of the Atacama Desert, Chile. Animals.

[23] Hernández-Martelo J., Jabs M., Contador T., Kim S., Lee S.y., Pérez L.M., Remedios-De-León M., Morelli E., Convey P., Benítez H.A. (2025). Evolving to invade: Using geometric morphometrics to assess wing shape variation in the Antarctic non-native fly Trichocera maculipennis. Zool. Anz..

[24] Hernández-Salinas U., Ramírez-Bautista A., Pavón N.P., Rosas Pacheco L.F. (2014). Morphometric variation in island and mainland populations of two lizard species from the Pacific Coast of Mexico. Rev. Chil. Hist. Nat..

[25] Milá B., Warren B.H., Heeb P., Thébaud C. (2010). The geographic scale of diversification on islands: Genetic and morphological divergence at a very small spatial scale in the Mascarene grey white-eye (Aves: *Zosterops borbonicus*). BMC Evol. Biol..

[26] Laojun S., Chaiphongpachara T. (2025). Phenotypic and genetic variation of *Aedes albopictus* (Diptera: Culicidae) in Thailand and its global relationships: Insights from wing morphometric and mitochondrial COI gene analyses. Med. Vet. Entomol..

[27] Benítez H.A., Pizarro-Araya J., Bravi R., Sanzana M.-J., Alfaro F.M. (2014). Morphological variation on isolated populations of *Praocis* (*Praocis*) *spinolai*. J. Insect Sci..

[28] Cameron S.A. (2004). Phylogeny and biology of neotropical orchid bees (Euglossini). Annu. Rev. Entomol..

[29] Khademi T.G. (2018). New insight into the phylogeny of the orchid bees (Apidae: Euglossini). J. Wildl. Biodivers..

[30] Ayala R., Gonzalez V.H., Ms E. (2022). The first pacific insular orchid bee (Hymenoptera, Apidae): A new species of Eufriesea from Islas Marias. J. Hymenopt. Res..

[31] Rojas B., Vásquez O., Santos-Murgas A., Cobos R., Gómez Robles I.Y. (2022). Abejas de las orquídeas como bioindicadores del estado de conservación de un bosque. Manglar.

[32] Costa C.P., Machado C.A.S., Santiago W.M.S., Dallacqua R.P., Garófalo C.A., Francoy T.M. (2020). Biome variation, not distance between populations, explains morphological variability in the orchid bee *Eulaema nigrita* (Hymenoptera, Apidae, Euglossini). Apidologie.

[33] Ferronato M.C.F., Giangarelli D.C., Mazzaro D., Uemura N., Sofia S.H. (2018). Orchid Bee (Apidae: Euglossini) Communities in Atlantic Forest Remnants and Restored Areas in Paraná State, Brazil. Neotrop. Entomol..

[34] Benítez H.A., Püschel T.A. (2014). Modelando la Varianza de la Forma: Morfometría Geométrica Aplicaciones en Biología Evolutiva. Int. J. Morphol..

[35] Toro Ibacache M.V., Manriquez Soto G., Suazo Galdames I. (2010). Morfometría geométrica y el estudio de las formas biológicas: De la morfología descriptiva a la morfología cuantitativa. Int. J. Morphol..

[36] Adams D.C., Rohlf F.J., Slice D.E. (2013). A field comes of age: Geometric morphometrics in the 21st century. Hystrix Ital. J. Mammal..

[37] Rohlf F.J., Slice D. (1990). Extensions of the Procustes methods for the optimal superimposition of landmarks. Syst. Zool..

[38] Klingenberg C.P. (2013). Visualizations in geometric morphometrics: How to read and how to make graphs showing shape changes. Hystrix Ital. J. Mammal..

[39] Klingenberg C.P. (2002). Morphometrics and the role of the phenotype in studies of the evolution of developmental mechanisms. Gene.

[40] Le Roy C., Debat V., Llaurens V. (2019). Adaptive evolution of butterfly wing shape: From morphology to behaviour. Biol. Rev..

[41] Castillo-Caballero P.L., Monteza-Moreno C.M., Johnson O., Angehr G.R. (2020). First annotated checklist of birds of Jicarón and Jicarita: The southernmost islands of the Republic of Panama. Tecnociencia.

[42] Castroviejo S., Velayos M. (1997). Flora y Fauna del Parque Nacional de Coiba (Panamá): Inventario Preliminar.

[43] Pérez R., Condit R., Aguilar S., Hernández A., Villareal A. (1996). Inventario de la vegetación de la isla de Coiba, Panamá: Composición y florística. Rev. Biol. Trop..

[44] Roubik D., Hanson P. (2004). Orchid Bees of Tropical America: Biology and Field Guide.

[45] Rohlf F.J. (2015). The tps series of software. Hystrix.

[46] Jolliffe I.T. (2002). Principal Component Analysis.

[47] Campbell N.A., Atchley W.R. (1981). The Geometry of Canonical Variate Analysis. Syst. Zool..

[48] Klingenberg C.P. (2011). MorphoJ: An integrated software package for geometric morphometrics. Mol. Ecol. Resour..

[49] Adams D.C., Otárola-Castillo E. (2013). Geomorph: An R package for the collection and analysis of geometric morphometric shape data. Methods Ecol. Evol..

[50] Smouse P.E., Long J.C., Sokal R.R. (1986). Multiple regression and correlation extensions of the Mantel test of matrix correspondence. Syst. Zool..

[51] Wang X., Que P., Heckel G., Hu J., Zhang X., Chiang C.-Y., Zhang N., Huang Q., Liu S., Martinez J. (2019). Genetic, phenotypic and ecological differentiation suggests incipient speciation in two Charadrius plovers along the Chinese coast. BMC Evol. Biol..

[52] Perrard A., Baylac M., Carpenter J.M., Villemant C. (2014). Evolution of wing shape in hornets: Why is the wing venation efficient for species identification?. J. Evol. Biol..

[53] Li H., Nabawy M.R.A. (2022). Wing Planform Effect on the Aerodynamics of Insect Wings. Insects.

[54] Kingsolver J.G., Koehl M.A.R. (1985). Aerodynamics, thermoregulation, and the evolution of insect wings: Differential scaling and evolutionary change. Evolution.

[55] Garzón M.J., Schweigmann N. (2018). Wing morphometrics of Aedes (Ochlerotatus) albifasciatus (Macquart, 1838) (Diptera: Culicidae) from different climatic regions of Argentina. Parasites Vectors.

[56] Wilk-da-Silva R., de Souza Leal Diniz M.M.C., Marrelli M.T., Wilke A.B.B. (2018). Wing morphometric variability in *Aedes aegypti* (Diptera: Culicidae) from different urban built environments. Parasites Vectors.

[57] Cook J.M., Crozier R.H. (1995). Sex determination and population biology in the hymenoptera. Trends Ecol. Evol..

[58] Heimpel G.E., de Boer J.G. (2008). Sex determination in the hymenoptera. Annu. Rev. Entomol..

